# Step-to-Step Ankle Inversion/Eversion Torque Modulation Can Reduce Effort Associated with Balance

**DOI:** 10.3389/fnbot.2017.00062

**Published:** 2017-11-14

**Authors:** Myunghee Kim, Steven H. Collins

**Affiliations:** ^1^Experimental Biomechatronics Laboratory, Mechanical Engineering, Carnegie Mellon University, Pittsburgh, PA, United States; ^2^Robotics Institute, Carnegie Mellon University, Pittsburgh, PA, United States; ^3^Department of Mechanical Engineering, Stanford University, Stanford, CA, United States

**Keywords:** prosthetics, locomotion, medical robots and systems, rehabilitation robotics, balance, stability

## Abstract

Below-knee amputation is associated with higher energy expenditure during walking, partially due to difficulty maintaining balance. We previously found that once-per-step push-off work control can reduce balance-related effort, both in simulation and in experiments with human participants. Simulations also suggested that changing ankle inversion/eversion torque on each step, in response to changes in body state, could assist with balance. In this study, we investigated the effects of ankle inversion/eversion torque modulation on balance-related effort among amputees (*N* = 5) using a multi-actuated ankle-foot prosthesis emulator. In stabilizing conditions, changes in ankle inversion/eversion torque were applied so as to counteract deviations in side-to-side center-of-mass acceleration at the moment of intact-limb toe off; higher acceleration toward the prosthetic limb resulted in a corrective ankle inversion torque during the ensuing stance phase. Destabilizing controllers had the opposite effect, and a zero gain controller made no changes to the nominal inversion/eversion torque. To separate the balance-related effects of step-to-step control from the potential effects of changes in average mechanics, average ankle inversion/eversion torque and prosthesis work were held constant across conditions. High-gain stabilizing control lowered metabolic cost by 13% compared to the zero gain controller (*p* = 0.05). We then investigated individual responses to subject-specific stabilizing controllers following an enforced exploration period. Four of five participants experienced reduced metabolic rate compared to the zero gain controller (−15, −14, −11, −6, and +4%) an average reduction of 9% (*p* = 0.05). Average prosthesis mechanics were unchanged across all conditions, suggesting that improvements in energy economy might have come from changes in step-to-step corrections related to balance. Step-to-step modulation of inversion/eversion torque could be used in new, active ankle-foot prostheses to reduce walking effort associated with maintaining balance.

## 1. Introduction

Individuals with below-knee amputation expend more energy during walking than their able-bodied counterparts (Waters and Mulroy, 1999). This increased effort may be partially due to an increased effort to maintain balance, and not to fall, especially when walking on challenging terrain (Paysant et al., 2006). For non-amputees, gait conditions that challenge balance can increase metabolic energy consumption, even though average gait mechanics remain similar (O'Connor et al., 2012; Voloshina et al., 2013). Changes are typically observed in step-to-step gait characteristics, such as increased step width variability and center of pressure variability. The energy used to perform these types of step-to-step corrective actions can be defined as balance-related effort, the portion of energy used to maintain balance, accounting for changes in energy cost that occur even while average mechanics remain unchanged. Just as external disturbances can increase balance-related effort, external assistance can reduce balance-related effort for both individuals with deficits (IJmker et al., 2013; IJmker et al., 2014) and non-disabled individuals (Donelan et al., 2004).

Passive ankle inversion/eversion compliance has been implemented in many ankle-foot prostheses, mostly to accommodate uneven ground (Lindhe, 2014; Childers et al., 2015). However, the effects of passive compliance on foot placement effort have been inconclusive (Childers et al., 2015) and have not resulted in reduced balance-related energy cost (Kim, 2015). Perhaps active inversion/eversion control may be more effective at reducing effort.

Active step-to-step control has proved useful for assisting with balance in studies with mathematical models and robotic devices, developed based on a cyclic, limit cycle walking approach. In this control approach, a state is sampled once per step, and the difference between the sampled state and the nominal state is calculated. The difference, or error, is used to decide the control action for the ensuing step. This approach has been successfully used to stabilize walking models (Kuo, 1999; Hobbelen et al., 2008) and robots (Bhounsule et al., 2014). Similarly, we previously used a dynamic model of three-dimensional limit cycle walking to develop controllers for an ankle-foot prosthesis. In the simulation study, we found that push-off work could be an effective control input for the step-to-step controller, allowing regulation of medio-lateral states (Kim and Collins, 2017), which are known to be the least stable (Kuo, 1999). When this step-to-step ankle push-off work modulation was implemented in an ankle-foot prosthesis, simulated amputees experienced reduced balance-related effort, indicated by decreased metabolic cost, reduced foot placement variability, and reduced mediolateral center of pressure variability (Kim and Collins, 2015). Our simulation study also showed that ankle inversion/eversion torque modulation could help restore balance under some circumstances (Kim and Collins, 2017). Step-to-step ankle inversion/eversion control could therefore be another balance assistance method suitable for ankle-foot prostheses.

The effects of prosthesis control on balance can be indicated by a combination of balance-related outcomes, including mechanical measures that often correlate with reduced metabolic cost (Donelan et al., 2004; IJmker et al., 2013; IJmker et al., 2014). Reductions in foot placement effort are indicated by reduced step width variability (Donelan et al., 2004; IJmker et al., 2014). A lessening of active ankle inversion/eversion control effort in the intact limb are indicated by reduced center of pressure variability on this side (Hof et al., 2007). Reduced step width can indicate increased balance confidence (Maki, 1997; Sheehan et al., 2016). An overall reduction in balance-related effort can be indicated by reduced metabolic cost (Donelan et al., 2004; IJmker et al., 2013) and by the user's subjective assessment (Esguerra and Johnson, 2016; Kalron et al., 2016), provided that changes in the prosthesis affect only step-to-step mechanics related to balance and not average prosthesis mechanics (Kim and Collins, 2015).

In this study, we explored the effects of step-to-step modulation of ankle inversion/eversion torque on balance through experiments with participants with below-knee amputation. We hypothesized that stabilizing step-to-step ankle inversion/eversion torque modulation in a robotic ankle-foot prosthesis would meaningfully contribute to overall balance maintenance, thereby reducing balance-related effort for the user. We also hypothesized that stabilizing prosthesis control would reduce balance-related effort while the same condition with opposite gain would have a destabilizing effect and increase effort. We expect the results of this study to offer insights into control methods for commercial prostheses that reduce balance-related effort for individuals with lower-limb amputation.

## 2. Methods

We implemented a controller that changes ankle inversion/eversion torque at each step while maintaining constant average torque and work in an ankle-foot prosthesis emulator. The effect of the controller on balance-related effort, indicated primarily by metabolic rate, was investigated in experiments with participants with unilateral, transtibial amputation.

### 2.1. Prosthesis control

#### 2.1.1. Emulator

**Figure 1:**
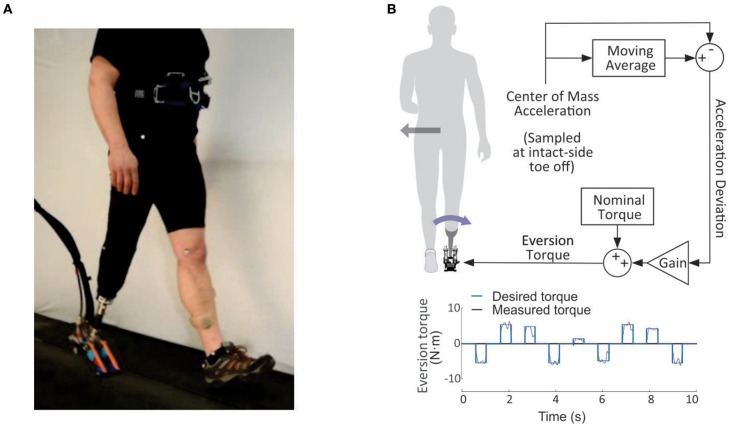
**(A)** Once-per-step ankle inversion/eversion controller. At the moment of intact-limb toe-off, prosthesis inversion/eversion torque was set for the ensuing stance phase. The torque was calculated by adding a nominal value, set for each participant on their preference, to the change in inversion/eversion torque. The change in inversion/eversion torque was equal to a gain times the difference between center-of-mass acceleration at the instant of toe off and a moving average of acceleration from the previous ten walking strides. With stabilizing control, an increase in center-of-mass acceleration could lead to a corrective eversion torque. An example pattern of eversion torques is shown at the bottom of the figure. The eversion torque was applied during each stance phase (around 0.6 s), which applied at each stride (around 1.1 s) at the bottom of the figure. **(B)** Photograph of the experimental setup. A tethered ankle-foot prosthesis with independent control of inversion/eversion and plantarflexion torques was used to apply the step-to-step controllers during experiments.

We used an ankle-foot prosthesis with control of both plantarflexion and inversion/eversion torques to test the effects of once-per-step inversion/eversion torque modulation on below-knee amputees (Figure 1A). This device had two independently actuated toes (described in detail in Collins et al., 2015). The mechanism provided inversion torque when the force of the outer toe was higher than that of the inner toe. By powering with two off-board motors, this testbed had a worn mass of only 0.72 kg, but it presented a plantarflexion torque of up to 180 N·m and an ankle inversion/eversion torque of up to ± 30 N·m, with a peak power of up to 3 kW. Ankle inversion/eversion torque limits were coupled to plantarflexion torque magnitude, but during most of the stance phase the allowable inversion/eversion torque was higher than seen in the biological ankle (Collins et al., 2015). The prosthesis also had a closed-loop torque bandwidth of higher than 20 Hz. Leveraging these characteristics, we were able to robustly test the desired controllers over a wide range of operating parameters.

#### 2.1.2. Controller design

##### 2.1.2.1. High-level, step-to-step control

The step-to-step controller chose an ankle inversion/eversion torque on each step as a linear function of the estimated medio-lateral acceleration at the beginning of each single-support period (Figure 1B). The control decision occurred at the instant of toe-off of the intact limb.

(1) $$\tau_{ev}=\tau_{nom}+K\cdot (a_{ref}-a_{ml})$$

where τ_*ev*_ is the desired ankle eversion torque for this step, τ_*nom*_ is the nominal ankle eversion torque, or a nominal torque, (a subject-specific torque, selected based on user preference during fitting and tuned at the beginning of each day as necessary), *K* is the control gain, *a*_*ml*_ is the lateral acceleration, and *a*_*ref*_ is the reference lateral acceleration calculated as a moving average over 10 steps. We limited τ_*ev*_ to ± 20 N·m, with a lower and upper limit set for any participant who experienced discomfort at higher inversion/eversion torque. Medio-lateral acceleration was determined using lateral force information from an instrumented treadmill (Bertec Co. Columbus, OH, USA). The value was obtained by dividing the sum of right and left lateral forces by the participant's mass. This was done for two reasons: to use the same calculations for both left- and right-side amputees, and to account for steps on which participants contacted both left and right halves of the treadmill. For the latter case, we detected single support using the emulator. When the emulator detected a force larger than 5 Nm, this was taken as an indication that foot flat had occurred and the single-support phase had been entered.

##### 2.1.2.2. Low-level, continuous control

The low-level controller in the ankle-foot prosthesis emulator calculated and controlled torque for each toe to meet the desired ankle inversion/eversion torque and plantarflexion torque. Desired inversion/eversion torque was commanded by the high-level controller and desired plantarflexion torque was calculated using a piecewise linear approximation of the human ankle work loop (Caputo and Collins, 2013). Ankle angle was determined by encoders affixed to each toe of the prosthesis. Heel-strike of the prosthetic limb was determined using both strain gages affixed to the fiberglass heel and force data from the instrumented treadmill to ensure accurate detection. Strain data from the heel of the prosthesis helped to filter out cases where the sound limb stepped on the prosthetic limb side of the treadmill. Ankle inversion/eversion and plantarflexion torques were measured using strain gages affixed to the top and bottom of each toe, with torque measurements calibrated using linear regression (Collins et al., 2015).

### 2.2. Experimental protocol

#### 2.2.1. Participants

Five individuals with below-knee amputation participated in this study (*N* = 5, all male, 4 traumatic and 1 dysvascular, all K3 ambulators, 3 left-side amputation, age = 47.8 ± 14.3 years, body mass = 88.6 ± 5.5 kg, height = 1.71 ± 0.037 m, time since amputation = 12.1 ± 7.4 years, mean ± s.d.). All participants had participated in previous studies with this device (Kim, 2015). The experimental protocol was approved by the Carnegie Mellon University Institutional Review Board and all experiments were conducted under these guidelines.

#### 2.2.2. Experimental protocols

We designed both a conventional group experiment and a single-case experiment to account for high inter-subject variability and the small number of participants. When high inter-subject variability exists, studying an individual's response to a controller may also help demonstrate the efficacy of the controller. Single-case experimental design can be used for such purposes (Dermer and Hoch, 2012). The predetermined order of a single-case experiment, however, may cause an ordering effect, which may impair the reliability and significance of the test. One solution is to apply the same conditions multiple times and average the results (Gentile and Klein, 1972). When a significant ordering effect presents, a more rigorous method needs to be employed (Hartmann, 1974) such as statistical analysis of single-case experiments with a random order (Edgington, 1967). This method, however, requires multiple evaluations of proposed conditions, which prolongs experimental periods. As a compensation method, we additionally investigated randomized group experiment results for each individual on top of the single-case experiment. The group experiment results also have used to evaluate whether the controller would be useful for another participant apart from the subject who showed a significance results.

**Figure 2:**
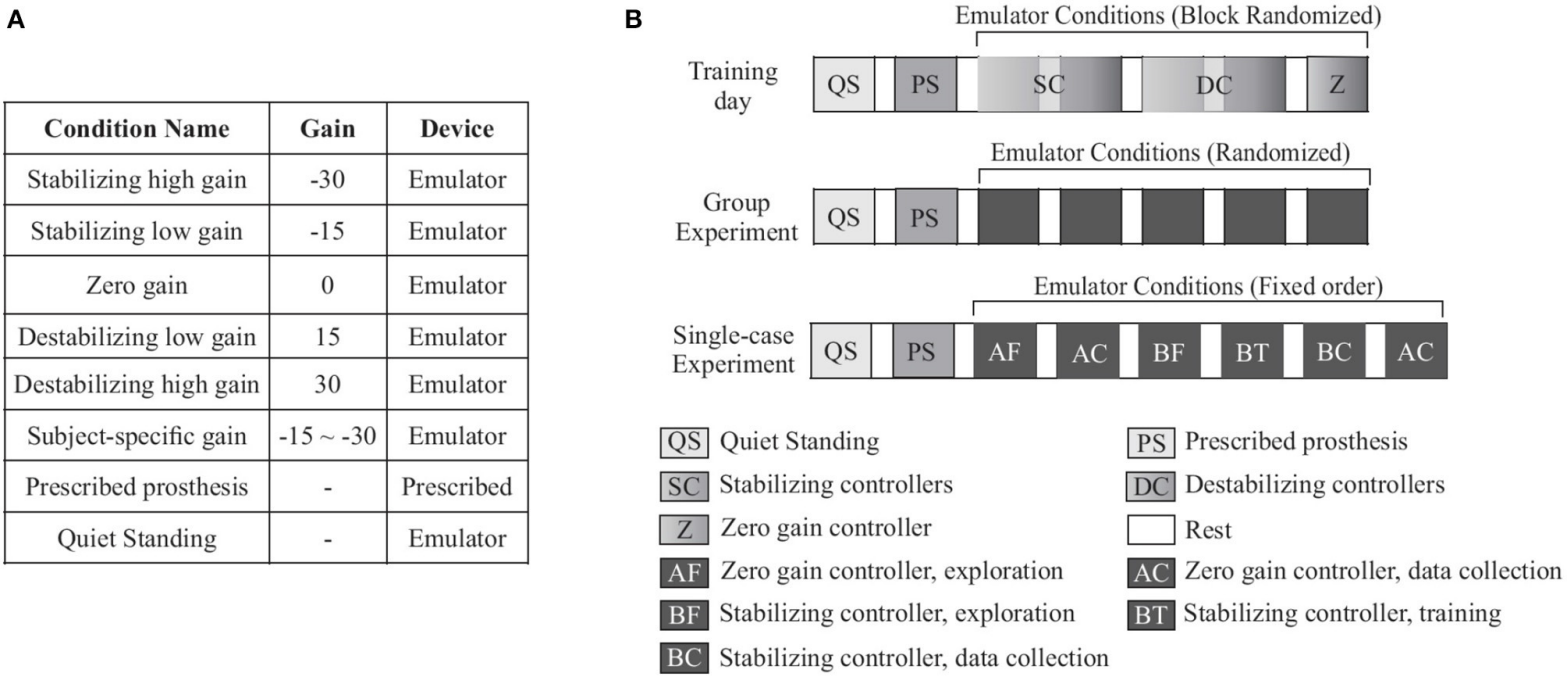
**(A)** Training and group experiment days included seven conditions, five with different inversion/eversion torque control gains and two baseline conditions. In the prescribed prosthesis condition, participants walked in their prescribed prosthesis. In the quiet standing condition, participants stood still while wearing the ankle-foot prosthesis emulator. **(B)** Each subject participated in one training day followed by a group experiment day and a single-case experiment day. On each day, participants first performed quiet standing. The prescribed prosthesis condition was randomized to occur either after quiet standing or at the end of the trial. During the training period, stabilizing controllers (SC), destabilizing controllers (DC), and zero gain (Z) were presented in random order. Stabilizing/destabilizing controller blocks were composed of the low and high gain controller, in that order. During the group experiment, all controller conditions were presented in random order. The single-case experiment was composed of the zero gain (A) and the stabilizing controller condition (B) with a fixed order. Each condition was preceded by a period of forced exploration (AF, BF).

Accordingly, we designed an experimental protocol with three sessions on separate days: an acclimation day, a group experiment day, and a single-case experiment day. During the acclimation and group experiment days, participants experienced five controller conditions: two with stabilizing gains, two with destabilizing gains and one with zero gain. During the single-case experiment session, participants were exposed to two controller conditions: a subject-specific stabilizing controller and a zero gain controller (Figure 2B).

During all conditions, some amount of ankle inversion torque was applied during stance. The value of this default ankle inversion torque was limited to the range of −5 to 5 N·m (Equation 1, τ_*nom*_) and was determined for each participant by hand tuning and user feedback (Caputo, 2015). Values for plantarflexion stiffness and work were set in the same manner. At the beginning of each session, a brief parameter check was performed to re-familiarize the user and ensure that all device parameters were comfortable.

The default walking period for each condition was 5 min, with 4 min of rest between trials. The cost was typically estimated by averaging 1 min of data to account for the expected noise per breath and rates of 0.2–0.3 breaths per second (Lourens et al., 2000; Ogawa et al., 2006; Garde et al., 2014). Slow and non-linear mitochondrial dynamics (Wai and Langer, 2016) and long transit time require a 2 to 3 min warm-up period (Selinger and Donelan, 2014). Assuming that motor learning processes do not cause additional slow changes in metabolic rate, last 1 min data of 4 min of respiratory data can, therefore, be used to estimate energy expenditure (Galle et al., 2017). For less-active participants who were unable to sustain this walking speed, the maximum sustainable speed was determined during the acclimation day. The default treadmill speed was set to 1.25 m·s^−1^. For less-active participants who were unable to sustain this walking speed, the maximum sustainable speed was determined during the acclimation day. Two subjects walked at 0.70 and 0.75 m·s^−1^, respectively (K3 ambulators).

##### 2.2.2.1. Day 1 - acclimation day

On the first day of testing, seven conditions were presented: five controller conditions, one quiet standing condition, and one prescribed prosthesis condition as a baseline. The five controllers were composed of a stabilizing high gain controller, a stabilizing low gain controller, a zero gain controller, a destabilizing low gain controller, and a destabilizing high gain controller. The conditions were divided into three blocks: one block with the two stabilizing controllers, one block with the zero gain controller, and one block with the two destabilizing controllers. The order of the three controller blocks was randomized (Figure 2B). For each stabilizing and destabilizing controller block, we presented the low gain and high gain controllers in the order of increasing gain. The gains were −30 N·m of inversion torque per m·s^−2^ of lateral acceleration (−30 N·s^2^) for the stabilizing high gain controller, −15 N·s^2^ for the stabilizing low gain controller, 0 N·s^2^ for the zero gain controller, 15 N·s^2^ for the destabilizing low gain controller, and 30 N·s^2^ for the destabilizing high gain controller (Figure 2A). These gains were selected based on a pilot study, in which we found that a 30 N·s^2^ gain led to ankle inversion/eversion torque in the range of ±10 N·m, which is twice the range observed during normal walking (Hunt et al., 2001) but within the comfortable range of most participants.

##### 2.2.2.2. Day 2 - group experiment

The second day of testing consisted of the same walking conditions as the first session, but on this day the five controller conditions were fully randomized. Data from this collection were used to determine the subject-specific gain for the single-case experiment using a procedure described below (Figure 2).

##### 2.2.2.3. Day 3 - single-case experiment

For the final day of testing, participants experienced eight conditions: six controller conditions, one quiet standing condition, and one condition with their prescribed prosthesis. For the six controller conditions, we presented the zero-gain controller (A) and subject-specific stabilizing controller with predetermined gain (B) repeatedly with the following order: A with enforced exploration for 4 min, A with data collection for 5 min, B with enforced exploration for 4 min, B with self-selected gain for 4 min, B with data collection for 5 min, and A with data collection for 5 min (AABBBA) (Figure 2).

##### 2.2.2.4. Gain selection

**Figure 3:**
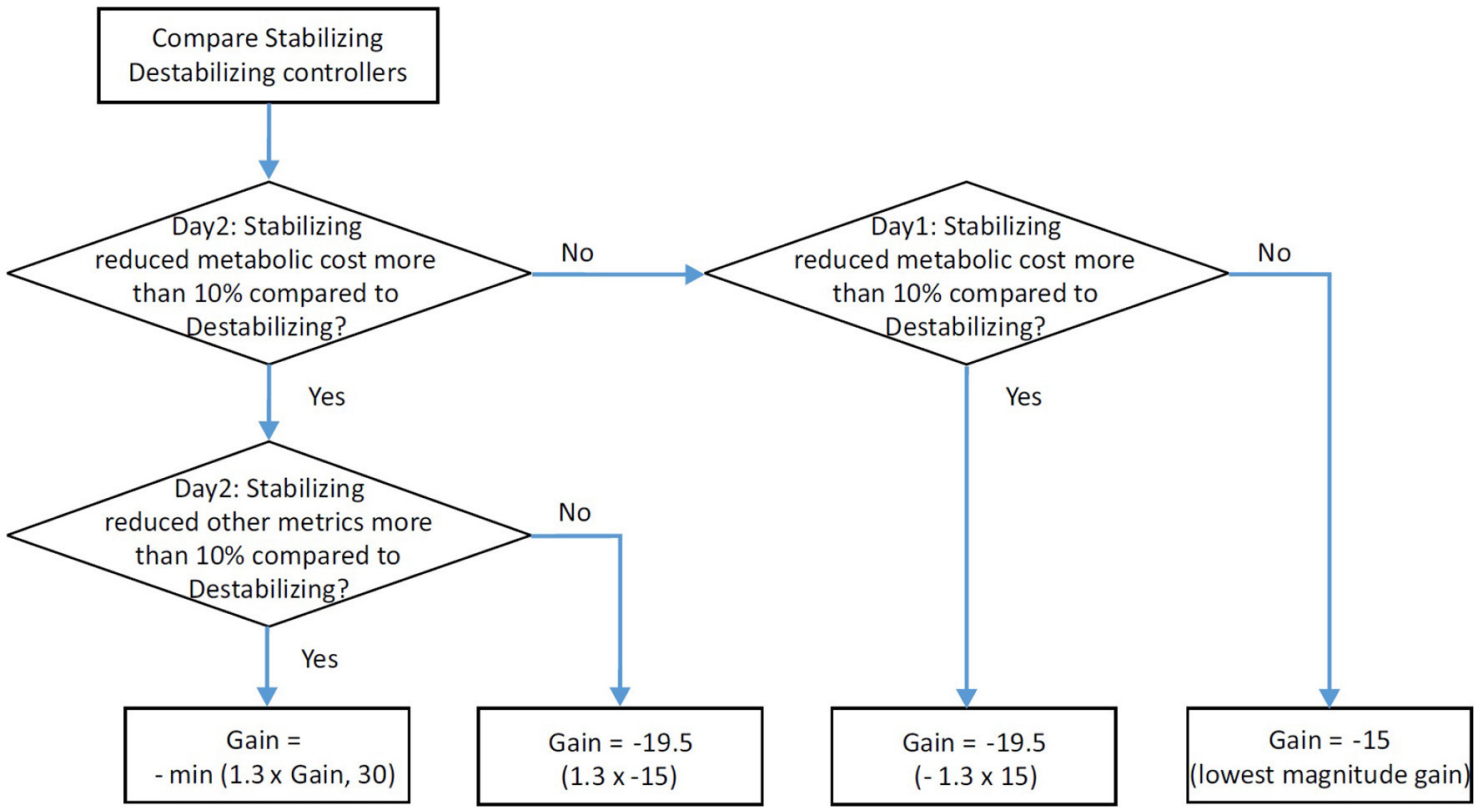
Flowchart for selecting the subject-specific gain used in single-case experiments.

Subject-specific stabilizing controller gains were selected based on the user's reaction to the gains applied in the group experiment (Figure 3). We compared the participant's response to the stabilizing and the destabilizing conditions. First, we examined the change in metabolic cost and other measures. If we found a 10% reduction in metabolic rate and a 10% improvement in one of the other indicators of balance-related effort (step width variability, average step width, center of pressure variability in the intact limb, or user preference), we concluded that the gain used in that condition was optimal. If this was the high gain, then we used that value. If this was the low gain, then we used a 30% higher gain for the experiment with enforced exploration, anticipating that a higher gain would be preferred after additional training. In the case that only metabolic rate showed more than a 10% reduction for the stabilizing controller condition compared to destabilizing controller condition, then we used a 30% higher gain than the lowest gain, based on pilot study results. If a trend was not clear from the group experiment, then we analyzed the acclimation period for that participant. Similarly, if we observed more than a 10% reduction in metabolic rate between the stabilizing controller condition and the destabilizing controller condition, then we applied a 30% higher gain than the low gain. If the participant did not show any reduction in metabolic rate, then we used the low gain. According to this process, −30 N·s^2^ was selected for one subject and −19.5 N·s^2^ for all other subjects.

##### 2.2.2.5. Enforced exploration

The effect of the controller could be clearly observed with intensive training. Amputees present higher inter-subject variability than their able-bodied counterparts (Quesada et al., 2016), partially due to differences in training periods (Zelik et al., 2011). The 2 days of training typically applied in this type of intervention may not be sufficient for some participants to adapt to the device fully. By providing additional training periods, enforced exploration (Selinger et al., 2015), participants may be able to learn to use a controller more effectively.

We chose the instructions for the enforced exploration periods through pilot tests in which we asked participants to move in ways that evoke several balance strategies found from the literature (Donelan et al., 2004; Hof et al., 2007; IJmker et al., 2013; Kim and Collins, 2015). Individuals with below knee amputation were able to follow these instructions. Each instruction was given for 20 s. We verbally gave the instruction and also visually demonstrated the motion. Typically, after the enforced exploration period, subjects were able to walk with reduced effort. This exploration seemed to help participants to understand the controller, which was complicated compared to their prescribed devices.

During enforced exploration periods, participants were instructed to attempt different walking strategies for a total of 3 min. After self-selected walking for 1 min, 12 different instructions were given at 15 s intervals. The instructions included: “lean a little bit to your left,” “lean a little more to your right,” “sway more side to side,” “lean a little forward,” “lean a little backward,” “keep your body upright and use limited sway,” “take slightly wider steps,” “take slightly narrower steps,” “take slightly longer steps,” “take slightly shorter steps,” “swing your arms more than you typically would,” and “swing your arms less than you typically would.” For the less active amputees, the instructions “keep your body upright and use limited sway,” “take slightly shorter steps,” and “swing your arms less than you typically would” were omitted and instructions were given every 20 s, as opposed to 15 s. This was done in order to preserve the overall length of the trial and allow more time for these participants to develop their gait based on the instructions. Participants all followed the instructions well, and the enforced exploration seemed to help them learn how to use the prosthesis better.

### 2.3. Balance-related measures

In order to determine balance-related effort, average step width, step width variability, intact-limb center-of-pressure variability, metabolic rate, and user preference were calculated (Kim and Collins, 2015). Center-of-pressure variability was calculated using force and moment data from the force-plate instrumented split-belt treadmill. Data was sampled at 1,000 Hz and low-pass filtered at 100 Hz. Step width variability and average step width were obtained using recorded data from 7 motion capture cameras (Vicon, Oxford, UK) using five motion capture markers (left heel, right heel, left toe, right toe and sacrum). We used 100 steps for both measures (Collins and Kuo, 2013). Net average metabolic rate (Brockway, 1987) was calculated using oxygen consumption and carbon dioxide production information obtained from a mobile metabolic system (Oxycon Mobile, CareFusion, San Diego, CA, USA). User preference was taken on a scale from −10 to 10 for both balance and comfort (with 0 being equivalent to walking on a prescribed device, 10 being effortless walking and −10 being very difficult to walk). Detailed calculation methods for each balance-related measure is described in Kim and Collins (2015).

### 2.4. Data analysis

We examined the differences in step-to-step changes in ankle inversion/eversion torque across conditions while maintaining average torque by comparing the means and standard deviations of ankle inversion/eversion torque using two-way ANOVA analyses, treating the gain as a continuous variable. Because average ankle plantarflexion and inversion/eversion work can affect metabolic cost, we also investigated the mechanical work across participants using a two-way ANOVA. Work was calculated by integrating power in time. We also examined torque tracking performance by averaging root mean square (RMS) error between measured and desired ankle inversion/eversion torque during stance over 100 steps. Stance phase was determined when the plantarflexion torque was higher than 5 N·m for this calculation. We also investigated the maximum and minimum ankle inversion/eversion torque.

External validity can be evaluated using both single-case and group experimental results. Single-case experiments can reveal the significance and reliability of an intervention to a participant as well as applicability to other participants by analyzing multiple participants' results (Barger-Anderson et al., 2004). We ran paired *t*-tests comparing the stabilizing controller condition to the average of the zero-gain conditions with a significance level α = 0.05 across subjects for all variables. We compared to the zero-gain condition, rather than prescribed, in order to minimize training effects, which can be important (Zelik et al., 2011) because participants have multiple years of experience with their prescribed passive ankle-foot prosthesis. We secondarily compared the stabilizing controller condition to the prescribed prosthesis condition using a paired *t*-test in order to investigate benefits that might be obtained despite having substantially less training with the emulator.

Generalizability could be bolstered by evaluating results from multiple participants in randomized trials to address ordering effects (Kim, 2015). In the group experiment, we tested for an effect of controller gain on each balance-related outcome by conducting a two-way ANOVA, treating the gain as a continuous variable. This approach was consistent with the experimental design, in which we obtained one replication for each combination of participant and controller condition. We used a linear model with a significance level α = 0.05. In cases where statistical significance was found, we further conducted a paired *t*-test by comparing the high-gain stabilizing controller to each of the controller conditions. We also performed a post-hoc power analysis. In addition, we examined the acclimation day results using the same method.

The effect of the subject-specific stabilizing controller on each participant was evaluated by investigating the single-case experiment results in terms of significance and reliability. The controller was considered to moderately or significantly lower balance-related effort if the difference between the zero-gain controller condition and the stabilizing controller condition was higher than 5 or 10%, respectively, based on previous results (Kim and Collins, 2015). For this comparison, we averaged data from the two zero-gain condition presentations to reduce ordering effects (Gentile and Klein, 1972). The reliability was visually examined by investigating whether the stabilizing controller condition resulted in lower energy cost compared both the zero-gain controller conditions (Dermer and Hoch, 2012). In addition, we also compared the stabilizing controller condition to the prescribed controller condition. The combined results speak to external validity to other participants and reliability and significance for each participant, strengthening our understanding of the effects of the controller on balance.

## 3. Results

### 3.1. Prosthesis mechanics

The controller provided stabilizing or destabilizing inversion/eversion torque once per step as a function of controller condition (ANOVA, *p* < 0.01) while maintaining constant average torque (ANOVA, *p* > 0.5). Net mechanical work due to both plantarflexion and inversion/eversion actuation did not differ across conditions (*p* > 0.1), as shown in Tables 1, 2. These results suggest that step-to-step changes in inversion/eversion torque influenced the biomechanics measures, not average ankle inversion/eversion torques or work. The controller tracked the desired inversion/eversion torque with a root mean square (RMS) error of about 1 N·m. The nominal torque, based on user preference at the beginning of each session, was 0.4 ± 0.9 N·m for the group experiment and 0.8 ± 1.1 N·m for the single-case experiment. Two participants requested a reduced range of torque, limited to [−1 5] and [−4 4] N·m, due to discomfort with larger torques. The Tables 1, 2 show the average, standard deviation, range, and RMS error of inversion/eversion torque for the group experiment and single-case experiment, respectively. Inversion/eversion torque was limited at the beginning of stance when the plantarflexion torque was low, which resulted in slightly lower average inversion/eversion torque than the nominal torque. Tracking performance was worst at the very beginning and very end of the stance phase, near changes in toe contact. When excluding those phases, the error was reduced by one-third (0.28 N·m on average).

**Table 1:** Prosthesis mechanics: Group experiment.

**Condition**	**Stab. high**	**Stab. low**	**Zero gain**	**Destab. low**	**Destab. high**
Average inversion/eversion torque (N·m)	0.299	0.316	0.373	0.347	0.315
Standard deviation inversion/eversion torque (N·m)	1.877	1.442	0.788	1.382	1.927
Minimum inversion/eversion torque (N·m)	−3.567	−2.555	−1.086	−2.735	−3.726
Maximum inversion/eversion torque (N·m)	4.458	4.167	1.737	4.038	4.806
RMS inversion/eversion torque error (N·m)	0.931	0.934	0.898	0.948	0.945
Average plantarflexion work (J)	5.671	5.780	5.690	5.625	5.904
Average inversion/eversion work (J)	0.021	−0.009	−0.010	0.006	0.006

**Table 2:** Prosthesis mechanics: Single-case experiment.

**Condition**	**Zero gain Exploration**	**Zero gain Data collection**	**Stabilizing Exploration**	**Stabilizing Training**	**Stabilizing Data collection**	**Zero gain Data collection**
Average inversion/eversion torque (N·m)	0.910	0.843	0.657	0.605	0.619	0.837
Standard deviation inversion/eversion torque (N·m)	0.905	0.803	2.085	1.762	1.755	0.704
Minimum inversion/eversion torque (N·m)	−0.679	−0.590	−3.883	−3.298	−3.114	−0.798
Maximum inversion/eversion torque (N·m)	2.315	2.001	4.814	4.507	4.448	1.827
RMS inversion/eversion torque error (N·m)	1.055	0.963	1.119	0.957	0.923	0.847
Average plantarflexion work (J)	3.581	5.116	4.452	5.510	5.440	5.334
Average inversion/eversion work (J)	−0.014	−0.004	−0.035	0.011	0.006	−0.003

### 3.2. Balance-related outcomes

**Figure 4:**
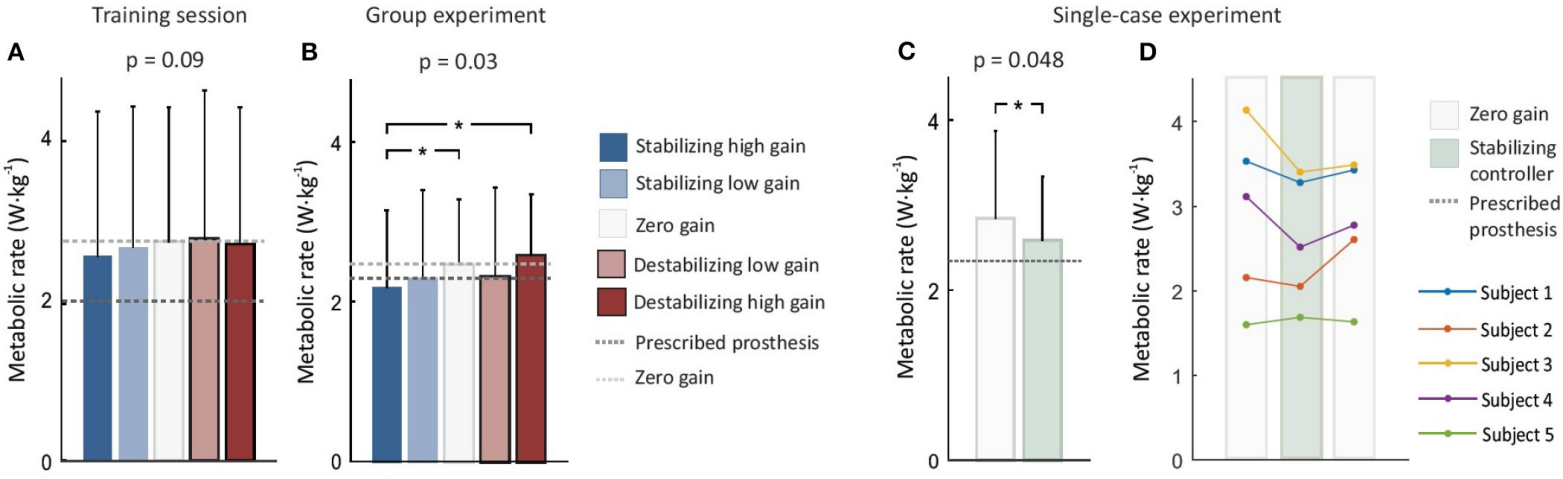
Metabolic rate across sessions. Bar graph shows average across subjects and error bars show standard deviation. **(A)** Training session results on day 1. Subjects showed a trend of lowered metabolic rate with the stabilizing controller. **(B)** Group experiment results on day 2. Subjects experienced reduced metabolic rate with the stabilizing high gain controller compared to the zero gain and destabilizing high gain controllers (^*^p < 0.05). **(C)** Single-case results on day 3. When the averaged zero-gain conditions and the subject-specific stabilizing controller conditions were compared, metabolic energy consumption was improved by 9% (^*^p < 0.05). **(D)** When each subject's response was examined, four subjects repeatably reduced their metabolic rate with the stabilizing controller.

In the single-case experiment, the subject-specific stabilizing controller reduced participant metabolic rate by 9% on average compared to the zero gain controller (*p* = 0.048; Figure 4C). Four participants experienced a reduction in metabolic rate (6, 11, 14, and 15%) and one participant had an increase in metabolic rate (4%; Figure 4D). Using these data, the achieved statistical power to discriminate the observed average reduction was 62%. There was no significant difference in metabolic rate between the stabilizing controller condition and the prescribed prosthesis condition (*p* = 0.5).

In the group experiment, we also observed reduction in metabolic rate (ANOVA *p* = 0.03; Figure 4B). The stabilizing high gain controller lowered average metabolic rate by 13 and 18% compared to the zero gain and destabilizing high gain controllers, respectively (*p* = 0.03 and *p* = 0.04). The achieved statistical power to discriminate these reductions was greater than 75%. Five participants reduced metabolic cost by 5, 8, 13, 31, and 6% for the stabilizing high gain controller compared to the zero gain controller. The destabilizing low gain controller seemed to lower metabolic cost by 6% compared to the zero gain controller but it was not statistically significant (*p* = 0.3). Metabolic rate seemed to be affected by controller gain during the acclimation day, but this effect was not statistically significant (ANOVA, *p* = 0.09; Figure 4A). Metabolic rate with the stabilizing high gain controller seemed to be about 4% lower than with participants' prescribed prosthesis, but this result was not statistically significant (*p* = 0.3).

In the single-case experiment (day 3), participants seemed to feel more comfortable and balanced with the stabilizing controller, although the experiment lacked sufficient statistical power to test this effect. Participants rated their balance with the stabilizing and zero-gain controllers as 0.6 ± 1.5 and 0.2 ± 1.6 (*p* = 0.2) and their comfort as 0.6 ± 1.4 and 0.3 ± 1.5 (*p* = 0.1), respectively. Similar results were found in the group experiment (day 2), in which participants rated the stabilizing high gain controller and destabilizing controllers as −0.42 ± 1.8 and −1.89 ± 2.5 for their balance, and −0.3 ± 2.0 and −1.81 ± 2.6 for their comfort, respectively. These differences were also not statistically significant (*p* = 0.3 and 0.2, respectively).

For the single-case experiment and group experiment (day 3 and 2), step width variability, average step width, intact-limb center-of-pressure variability and perception of comfort did not appear to be affected by once-per-step inversion/eversion torque controller gain (*p* > 0.3).

On the acclimation day (day 1), stabilizing control gains resulted in improved perception of balance ability (ANOVA, *p* = 0.048). We also noticed that during the course of acclimation subjects seemed to slowly reduce step width variability, average step width and center of pressure variability.

## 4. Discussion

In this study, we tested the hypothesis that step-to-step modulation of prosthetic ankle inversion/eversion torque would meaningfully reduce balance-related effort among unilateral transtibial amputees. We found that this control approach substantially reduced metabolic rate compared to a zero gain control in both single-case and group experimental designs. This reduction in metabolic rate was due to step-to-step changes in inversion/eversion torque associated with balance, and not with average prosthesis behavior, since the average ankle inversion/eversion torque over many steps was constant across conditions.

To maintain balance while walking, gait changes are typically made at each step to correct deviations from the nominal pattern, and this control is associated with metabolic cost. Assistance can help reduce such step-to-step corrective effort, even when the nominal gait pattern is unchanged (Donelan et al., 2004; IJmker et al., 2013; Kim and Collins, 2015). In our study, the stabilizing controller reduced metabolic cost while other average mechanics were maintained. This reduction therefore most likely came from reduced step-to-step effort to maintain balance by utilizing ankle inversion/eversion corrective torque. This finding is consistent with a previous simulation study in which once-per-step inversion/eversion torque control assisted with balance maintenance. Beneficial effects seemed to be shown on the first day of acclimation (Figure 4A) and improved with additional exposure (Figures 4B–D).

The inversion/eversion torque modulation seemed easy enough to use; therefore, additional training through forced exploration seems not necessary. In our previous study, we found an opposite result: participants were able to use assistance, once-per-step modulation of ankle push-off work, only after the forced exploration. The method influenced side-to-side motion by changing push-off work (Kim, 2015). In contrast, the current method affected side-to-side motion by providing side-to-side torque (inversion/eversion torque) based on side-to-side motion deviation. This control action seemed to be understandable with minimal training.

Participants seemed to easily understand the control action, providing medio-lateral torques based on medio-lateral acceleration deviation from the nominal value, and reduce walking efforts, partially associated with balance. Several types of balance restoring methods exist, majorly foot placement and ankle inversion/eversion control, which can affect metabolic cost (Donelan et al., 2004; Hof et al., 2007; IJmker et al., 2013; Kim and Collins, 2015). Those balance restoring effort can be changed depending on age and walk speed (Hurt et al., 2010; Major et al., 2013). After the acclimation period, participants might have been able to learn to reduce the effort associated with a combination of balance strategies in addition to foot placement (Donelan et al., 2004; O'Connor et al., 2012), intact limb control effort (Hof et al., 2007), or upper body motion (Curtze et al., 2011; Riva et al., 2013; Beurskens et al., 2014). By measuring additional indicators of balance-related efforts, such as upper body involvement, we might be able to explain the causes of reduced metabolic rate. Testing more subjects would also help uncover a cause by increasing statistical power in the face of substantial measurement noise in metabolic rate measurement, which shows low signal-to-noise ratio.

Although the stabilizing controller reduced metabolic cost compared to the zero gain controller, the metabolic cost was higher than with participants' prescribed devices. One of the major reasons is likely the difference in training period (Zelik et al., 2011). Participants in this study had used their prescribed prosthesis for 12 years on average, but had used the emulator for less than a day. Further exposure to the stabilizing controller, for example in a portable device used for a few weeks, might yield stronger results. Improvements in other features of the emulator hardware, such as the shape and stiffness of the passive heel keel, might also lead to lower metabolic rate, independent of controller.

Metabolic rate appeared to be reduced more in the group experiment than in the single-case experiment unlike our hypotheses, subject-specific controller would further reduce metabolic cost, and forced exploration would help for participants use the assistance. This result could be explained by a gain selection process, fatigue, and the controller characteristics. For the single-case experiment on the day 3, we did not select high gain because only metabolic cost showed a reduction. If we had used the highest gain for all participants based on metabolic cost results, participants might have experienced further reductions in the balance-related effort. On the other hand, the intensive forced exploration period on the day 3 seemed to result in fatigue for at least one subject, indicated by the increased metabolic rate for all subsequent conditions. The metabolic cost might have been reduced more by improving the gain selection process and providing additional rest periods or days. Perhaps improved methods for enforced exploration could help participants to discover a way to use the proposed controller even more effectively (Selinger et al., 2015).

The interpretation of these results is limited due to the small number of participants tested. Furthermore, the participants in our study showed a wide range of age, years since amputation, and walking speeds, which also contribute high inter-subject variability (Hurt et al., 2010; Major et al., 2013). To help account for high inter-subject variability (Fey et al., 2010; Wentink et al., 2013), we investigated the effect of the controller on each individual as well as the group response. Even though we observed a meaningful reduction in metabolic cost from responders and external validity, the generalization could be strengthened by testing a larger number of participants. Larger group sizes may also help uncover the underlying mechanics of the observed metabolic cost reduction.

In this study, enforced exploration did not seem to provide a substantial benefit to participants. Reductions in metabolic rate seem to have been similar between group and single-case experiments. Perhaps improved methods for enforced exploration could help participants to discover a way to use the proposed controller even more effectively (Selinger et al., 2015).

Our simulation study suggests that ankle inversion/eversion torque control was better able to handle specific types of disturbances (Kim and Collins, 2017). Both the inversion/eversion and push-off work controllers show the capacity to reduce balance-related effort (Kim and Collins, 2017). Perhaps an appropriate combination of these controllers might further enhance balance and improve recovery from combined disturbances, including lateral impulses and ground height changes.

This study demonstrates that ankle inversion/eversion control can reduce balance-related effort during walking, as shown by a reduction in metabolic rate. The controller seemed to allow participants to reduce their metabolic energy consumption with relatively little training. The control behavior also seemed to help participants perceive its benefits easily, and led them to prefer the stabilizing controller over the destabilizing controller during their acclimation day. Although the underlying causes of the reduction in balance-related energy use are unclear, the repeated and substantial reduction in metabolic rate confirms that modulation of ankle inversion/everion torque can be a successful walking assistance method for an ankle-foot prosthesis.

## Author contributions

MK and SC designed the control strategy and human-subject experiment. MK conducted the experiment and analyzed the data. MK outlined and drafted manuscript and SC edited the manuscript.

### Conflict of interest statement

The authors declare that the research was conducted in the absence of any commercial or financial relationships that could be construed as a potential conflict of interest.
